# Predictors of long acting and permanent contraceptive methods utilization among Women in Rural North Shoa, Ethiopia

**DOI:** 10.1186/s40834-017-0049-2

**Published:** 2017-08-25

**Authors:** Fantahun Ayenew Mekonnen, Wassie Negash Mekonnen, Solomon Hailemeskel Beshah

**Affiliations:** 1 Department of Epidemiology and Biostatistics, Institute of Public Health, College of Medicine and Health Sciences, University of Gondar, Gondar, Amhara Regional State Ethiopia; 2 Department of Midwifery, Institute of Medicine and Health Sciences, Debre Berhan University, Debre Berhan, Amhara Regional State Ethiopia; 3 Department of Public Health Officer, Institute of Medicine and Health Sciences, Debre Berhan University, Debre Berhan, Amhara Regional State Ethiopia

**Keywords:** Married Women, Long acting and permanent contraceptive methods, Ethiopia

## Abstract

**Background:**

According to available evidence, one in three married women in Ethiopia tends to avoid multiple children. On the other hand, women using Long Acting and Permanent Contraceptive Methods (LAPMs) are just 5 %. So, we aimed at identifying the factors associated with the utilization of LAPMs.

**Methods:**

We conducted a community based unmatched case control study among married women living in the rural areas of North Shoa zone, Ethiopia, in March 2015. The cases were married women using LAPMs, while controls were married women who were using modern short term methods. We recruited a total sample of 406 married women for this study on a 1:1 case to control ratio basis. We collected the data through interview using a pre tested questionnaire, and then a logistic regression model was fitted to the data to examine factors associated with the utilization of LAPMs. Adjusted Odds Ratio (AOR) with the corresponding 95% confidence interval was computed.

**Results:**

In our study, women whose husbands were daily laborers [AOR; 95% CI: 4.4(1.23,15.72)], who had $85–$140 monthly household income [AOR; 95% CI: 1.8(1.10,3.14)], and who were aged less than 20 years and below when they gave the first birth [AOR; 95% CI: 1.78, 4.90) were more likely to use LAPMs compared to women whose husbands were government employees, who had less than $85 monthly household income, and who were aged 20 years and above when they gave first child.

**Conclusion:**

We found that husbands’ characteristics were more important than their wives characteristics in influencing women’s utilization of LAPMs though such husband characteristics considered in this study were few in number. So, we recommend further research to examine the different characteristics of husbands responsible for women’s utilization of LAPMs.

## Background

Population is still growing rapidly in some developing countries, especially in Africa although it has slowed down in the rest of the world. There is a rapid fall in the average number of children per woman in such developing countries, as China, India, Indonesia, Iran, Brazil, and South Africa, while rapid growth is expected to continue over the next few decades in countries with high levels of fertility such as Nigeria, Niger, the Democratic Republic of Congo, Ethiopia, Uganda, Afghanistan, and Timor-Leste, where the rate is more than five children per woman [1, 2]. Ethiopia has become the second most populous country in Africa with a population of about 90 million, projected to be 178 million by 2050 [3].

High parity restricts women’s educational and economic opportunities, thereby broadly limiting their potential for empowerment and their ability to safeguard the health and economic well-being of the family and the community at large. Moreover, high fertility affects the well-being of mothers and their children, increasing maternal morbidity and mortality [4].

According to Ethiopia’s Demographic and Health Survey (EDHS) report, maternal mortality ratio was 676 maternal deaths per 100,000 live births, which was different from what was released in the 2000 and 2005 EDHS [5]. In this regard, providing women with greater contraceptive choices needs to be an integral element of raising the overall levels of contraceptive use and reducing the rates of unwanted pregnancies, thereby reducing the risk of maternal morbidity and mortality [6–9].

In Ethiopia, more than 37% of married women wanted no more children, and the contraception discontinuation rate is 37% for all methods, largely due to short term methods which include pills (70%) and condoms (65%). However, women who are using Long Term and Permanent Contraceptives are only 5% [10].

A wide range of factors including women’s age, residence, number of living children, women’s and men’s education and working status, religion, desire for more children, visits by family planning workers and husbands’ views of family planning, and knowledge of men and women about contraceptives are the commonly reported characteristics responsible for women’s behavioral change towards the utilization of contraceptives [11–13].

To our knowledge, research has not been conducted to identify the underlying factors for women’s low utilization of Long Acting and Permanent Contraceptives in the study area so far. On the one hand, a reasonable number (one in three) of women demonstrate disinterest in having many children, and the rate of discontinuation of short term (pills and condoms) has been going on unabated. So, this study set out to examine the predictors of LAPM of contraception among rural women in North Shoa zone, Ethiopia.

## Methods

### Study design and setting

We conducted a community based unmatched case control study to identify factors associated with LAPMs utilization among married women in Basona Worana district, North Shoa zone, Ethiopia. Basona Worana district is one of the rural districts in North Shoa zone which has a total population of 134, 600 out of which 48.8% are females. The economic activity of 98% of the population of the district is agriculture.

### Case definitions

#### Cases

Married women who were using Long Acting and Permanent Methods of contraception, including Implant, IUD (Intra Uterine Device), and Sterilization during the data collection period.

#### Controls

Married women who were using modern short term methods, including pills, condoms, and injectables during the same period.

### Inclusion and exclusion criteria

All married women who were using modern family planning methods and have lived in the area at least for about 6 months were included, while married women with impaired hearing, and serious illness at the time of data collection were excluded.

### Sample size determination

We determined the sample size using the proportion of exposure among controls and effect size between the exposure and LAPMs use. Married women aged over 25 years among the short term family planning users were considered as exposed controls, and the Odds Ratio (OR) of the association between age of women and their LAPMs use was taken as the effect size. So, a proportion of 0.76 and an Odds Ratio of 2.3 were taken from a previous study [14]. We assumed 80% power, and 95% level of confidence. The sample size obtained using Epi info statcalc was 179 cases and 179 controls. After adding a 10% non – response rate, the total sample size became 394 based on a 1:1case to control ratio.

### Sampling procedure

First, we selected kebeles, the smallest administrative units in the hierarchy of government administration. The number of kebeles was determined in consultation with Health Extension Workers (HEW) in such a way that it was possible to obtain a minimum of 197 cases. Then, all 203 cases in the kebeles were included in the study. For every case, one married woman who was using modern short term methods and whose house was very proximate to the house of the case was selected as control to enhance comparability between cases and controls. In households with two or more eligible respondents, only one respondent was selected using the lottery method.

### Data collection

Data on married women were collected by interview using a structured questionnaire. The questionnaire was developed in English and then translated into Amharic. The data collectors were nurses who spoke the local language (Amharic). They collected the data after they were given training on data collection procedures to attain standardization and maximize interviewer reliability. They were supervised regularly by the investigators to ensure proper data collection.

### Data quality control

To maintain the quality of the data, the questionnaire was pretested, and the data collectors were given a 1 day training. The training focused on areas relating to the purpose of the study, the data collection tools, participant approach and handling, and information confidentiality, etc. The data collectors were closely supervised to ensure proper performance. Completeness and consistency of data were also checked hand in hand with the data collectors and supervisors for immediate correction.

### Data processing and analysis

We again checked the collected data for completeness and consistency, entered twice, and cleaned to avoid entry errors. The data were then described by computing means, proportions, and tables as deemed necessary. We performed bi-variable and multi-variable analysis using binary logistic regression, and computed adjusted odds ratios with the corresponding CI (Confidence Interval) and *P*-value. Tests for interaction, multi-colliniarity, and model fitness were carried out. Descriptive and analytic computations were performed using Stata version 12 software.

### Ethical consideration

Ethical approval was obtained from the Research Ethics Review Committee of the Institute of Medicine and Health Sciences, Debre Berhan University, and then a letter of cooperation was written to the respective study area administrative offices so that permission to approach the intended participants was obtained to carry out the actual study. During the actual data collection, there was a close supervision of data collectors to ensure the application of the fundamental research ethical principles, and rules which included informed decision for participation through detailed information provisions, privacy and confidentiality, etc.

## Results

In this study, a total of 392 married women, 203 of whom were not using LAPMs of contraception and considered as controls, and 189 who were using LAPMs and considered as cases completed the study. The mean age of LAPM of contraception user women was 32.34 with a 6.3 standard deviation, while the mean age of their counterparts was 30.17, with a 6.4 standard deviation.

### Socio demographic characteristics

Almost all of the participants were Amhara by ethnicity and Orthodox Christians. Over half, 107(56.61%), of the LAPMs users and 87(42.86%) of the non-users were above 30 years of age. Most of the women in each group reported that they were 20 years and below at marriage. Regarding schooling, 44 (23.28%) of the cases and 75(36.95%) of the controls had formal education. The main occupation of the husbands, 148 (78.3%), of the cases and 154 (75.86%), of the controls was farming. (Table 1)

**Table 1:** Socio-demographic characteristics of married women using modern contraceptives in North Shoa, Ethiopia, March, 2015

Variables	LAPMs use	
	Cases	Controls
	no(%)	no(%)
Age(in year)		
≤ 30	82(43.39)	116(57.14)
> 30	107(56.61)	87(42.86)
Age at marriage(in year)		
≤ 20	143(75.66)	158(77.83)
> 20	46(24.34)	45(22.17)
Family size		
1–2	17(9.29)	33(16.75)
3–4	66(36.07)	75(38.07)
≥ 5	100(54.64)	89(45.18)
Wife education		
No formal education	145(76.72)	128(63.05)
Formal Education	44(23.28)	75(36.95)
Husband education		
No formal education	145(76.72)	131(64.53)
Formal education	44(23.28)	72(35.47)
Wife occupation		
House wife	161(85.19)	163(80.30)
Daily laborer	25(13.23)	28(13.79)
Government employee	3(1.59)	12(5.91)
Husband occupation		
Government employee	6(3.17)	15(7.39)
Daily laborer	35(18.52)	34(16.75)
Farmer	148(78.31)	154(75.86)
Monthly income		
< $85	53(34.64)	87(48.88)
$85–$140	67(43.79)	63(35.39)
> $140	33(21.57)	28(15.73)

### Reproductive characteristics of respondents

Both cases and controls were also asked questions which were believed to make differences in terms of using LAPMs. Out of the cases and controls asked to report the number of live children they had, very few 5(2.76%) of cases and 11(5.76) of controls reported that they had no live children, while 29(15.34%) of cases and, 58(28.57%), of controls reported they had no pregnancy history. Twenty-seven (14.29%) of the cases and 20(9.85%) of the controls reported they had history of abortion (Table 2).

**Table 2:** Reproductive characteristics of married women using modern contraceptives in North Shoa, Ethiopia, March, 2015

Variables	LAPMs use	
	Cases	Controls
	no(%)	no(%)
Have alive child		
Not at all	5(2.76)	11(5.76)
1–2	64(35.36)	80(41.88)
3–4	66(36.46)	61(31.94)
≥ 5	46(25.41)	39(20.42)
Age when giving birth		
≤ 20	130(71.43)	97(53.59)
> 20	52(28.57)	84(46.41)
Previous pregnancy		
Yes	160(84.66)	145(71.43)
No	29(15.34)	58(28.57)
Encountered abortion		
Yes	27(14.29)	20(9.85)
No	162(85.71)	183(90.15)

### Factors associated with utilization of LAPMs

About 16 independent variables were examined for possible associations with LAPMs contraception use, using the multivariable logistic regression model. Among the variables treated by the multivariable analysis, variables which included husbands’ occupation, house hold monthly income, and age of the woman when she gave her first birth were statistically significant at alpha value of 0.05 adjusted for husband education and number of pregnancies, yielding a Hosmer and Lemeshow’s goodness of model fit test value (*p* = 0.37).

Women with a household monthly income of $85–$140 were 1.8 times more likely to use LAPMs compared with women whose household income was less than $85 [AOR (95% CI) = 1.8(1.07, 3.14)]. Women whose husbands were daily laborers were 4.4 times more likely to use LAPMs as compared with women whose husbands were government employees [AOR (95% CI) = 4.4(1.07, 3.14)]. On the other hand, women who gave the first birth at the age of 20 or less were 2.9 times more likely to use LAPMs compared with their above 20 years of age married counter-parts [AOR (95% CI) = 2.9(1.78, 4.90)].

Husbands’ education and number of pregnancies were found to be confounding variables in the association between husband’s occupation and women’s LAPMs use. When both husband’s education and number of pregnancy variables were simultaneously removed from the final model, a great change in the size of the odds ratio of the association between the variable husband’s occupation and LAPMs use was observed. When only husband’s education was removed from the model, besides the change in the size of the odds ratio of husband occupation, it left the variable number of pregnancy statistically significant and vice versa (Table 3).

**Table 3:** Factors associated with LAPMs utilization among women in North Shoa, Ethiopia, March, 2015

Variables	LAPMs use		COR (5% CI)	AOR (95% CI)
	Cases no (%)	Control no(%)		
Age when giving first birth^*^				
≤ 20	130(71.43)	97(53.59)	2.16 (1.40, 3.34)	2.9(1.78, 4.90)
> 20	52(28.57)	84(46.41)	1	1
Husband education				
No formal education	145(76.72)	131(64.53)	1	1
Formal education	44(23.28)	72(35.47)	0.55 (0.35, 0.86)	0.6(.33, 1.08)
Previous pregnancy				
Yes	160(84.66)	145(71.43)	2.20 (1.34, 3.64)	1.8(0.90, 3.48)
No	29(15.34)	58(28.57)	1	1
Income^**^				
> $85	53(34.64)	87(48.88)	1	1
$85–140	67(43.79)	63(35.39)	1.75 (.08, 2.83)	1.8(1.07, 3.14)
> $140	33(21.57)	28(15.73)	1.93 (1.05, 3.55)	1.96(0.99, 3.88)
Husband occupation^**^				
Government employee	6(3.17)	15(7.39)	1	1
Farmer	148(78.31)	154(75.86)	2.57 (0.89, 7.41)	1.9(0.60, 6.54)
Daily laborer	35(18.52)	34(16.75)	2.40 (0.91, 6.36)	4.4(1.23, 15.72)

^*^ *P*-value < 0.01; ^**^ *P*-value < 0.05

## Discussion

We aimed at finding out the factors associated with the utilization of Long Acting and Permanent Methods of contraception. The factors identified associated with LAPMs included, occupation of husband, household monthly income, and age of woman when she gave the first birth. These findings are similar to the findings of studies conducted in Tanzania, Indonesia, and Delhi, India [12, 13, 15].

In our study, women whose husbands were daily laborers were 4.4 times more likely to use LAPMs contraception compared with women whose husbands were government employees. This finding is consistent with the demographic and health survey reports of some countries where the odds of approving family planning are higher when the husband is employed [16]. This may be due to the fact that daily laborers are people with insecure jobs and unstable life who prefer long term family planning methods until they get jobs that guarantee consistent and continuous income generation. The other possible explanation may be the link that exists between occupation and education that when wives are more educated they are more likely to use short term methods as indicated by the finding of a study conducted in Batu town, Ethiopia, showing that spouses are more likely to jointly approve of family planning when the husband is better educated than his wife [16]. But they were less likely to use sterilization when the husband is less educated due to side effects and convenience considerations [17].

Women with house-hold monthly income of $85–$140 were 1.8 times more likely to use LAPMs compared with women whose house-hold income was less than $85; no difference was observed among women who had monthly income of more than $140 and less than $85. Available evidences on this issue are conflicting. The findings of one qualitative study revealed that poor women chose vasectomy on the ground that having more children affected the quality of life, making it difficult to buy even a soap [15]. Another study identified that wealthier households were more likely to reach spousal agreement on family planning than their counterparts [16].

Women who gave their first birth at age 20 or less were 2.9 times more likely to use LAPMs compared with their counter parts. This finding might be indirectly supported by the finding of a study conducted on spousal agreement on the approval of family planning that revealed a higher percentage of spousal agreement on approval of family planning among couples in which the wife’s age was15–34 compared with couples where the wife’s age was 35–49 [16].

In the current study, education of husband and number of pregnancies were not found to be statistically significantly associated, which is inconsistent with previous studies [16, 17], but they were identified as confounding variables in the association between occupation of husband and utilization of LAPMs. In addition, they were confounding variables for each other, meaning when one was removed from the final model, the remaining one became statistically significantly associated.

In our study, information related to husbands’ characteristics was limited; we collected data on a range of women’s characteristics, while we collected data on two husbands’ characteristics and one household characteristic. Of the two, one was significantly associated and the other was found to be a cofounding variable.

## Conclusion

We identified occupation of husband, household monthly income, and age of woman significantly associated with women’s utilization of LAPMs. We found that husbands’ characteristics were more important than the characteristics of the wives in influencing women’s’ utilization of LAPMs.

## Abbreviations

AOR: Adjusted odds ratio
CI: Confidence interval
COR: Crude odds ratio
CSA: Central statistical agency
EDHS: Ethiopian demographic and health survey
HEW: Health extension workers
IUD: Intra uterine device
LAPMs: Long acting and permanents methods
UN: United Nation
